# Cyclic Altitude Training, Mitochondrial Health, and the Oral–Airway Axis: Intermittent Hypoxia Between Adaptation and Disease

**DOI:** 10.3390/jcm15145402

**Published:** 2026-07-10

**Authors:** Mark Cannon, John Peldyak, Paul R. Reynolds, Benjamin Bikman

**Affiliations:** 1 Ann and Robert H. Lurie Children’s Hospital, Northwestern University, Chicago, IL 60611, USA; 2 Independent Researcher, Mt. Pleasant, MI 48858, USA; 3 Department of Cell Biology and Physiology, Brigham Young University, Provo, UT 84602, USA

**Keywords:** intermittent hypoxia, cyclic altitude training, mitochondria, obstructive sleep apnea, periodontitis, oral–systemic health, airway, PGC-1alpha, HIF-1alpha

## Abstract

Mitochondria regulate cellular energetics, redox balance, apoptosis, and inflammatory signaling in oral, airway, and systemic tissues. Hypoxia is a powerful modulator of mitochondrial function, with effects ranging from adaptive hormesis to overt injury. Cyclic altitude training, most often delivered as intermittent hypoxic exposure or intermittent hypoxia training (IHT), has been proposed as a strategy to improve mitochondrial efficiency and exercise performance. By contrast, obstructive sleep apnea (OSA) exposes patients to uncontrolled chronic intermittent hypoxia (CIH), a pattern increasingly linked to endothelial dysfunction, ceramide-mediated mitochondrial dysfunction, insulin resistance, systemic inflammation, oral dysbiosis, and periodontitis. This narrative review covers intermittent hypoxia, mitochondrial biogenesis, hypoxia-inducible factor signaling, OSA, periodontitis, oral microbiome shifts, nitric oxide biology, and smoke-related mitochondrial injury. Appropriately dosed IHT can increase mitochondrial biogenesis, improve mitochondrial morphology, and augment oxidative capacity through pathways involving PGC-1alpha, hypoxia-inducible signaling, mitochondrial dynamics, and reactive oxygen species-dependent hormesis. In contrast, CIH in OSA promotes oxidative stress, sympathetic activation, endothelial injury, and inflammatory signaling and is associated with worse periodontal status and altered salivary microbiome profiles. Controlled IHT and OSA-related CIH, therefore, represent opposite ends of a hypoxia continuum, and mitochondrial health connects sleep-disordered breathing, periodontal inflammation, environmental exposures, and systemic cardiometabolic risk within a single conceptual frame. Sphingolipid signaling—particularly hypoxia- and toxicant-driven ceramide accumulation—connects CIH, inhaled environmental exposures, mitochondrial fragmentation, and the development of insulin resistance.

## 1. Introduction

Mitochondria are central to ATP generation, redox signaling, innate immune responses, and programmed cell death across nearly all mammalian tissues. In the oral cavity and upper airway, mitochondrial integrity influences epithelial barrier function, fibroblast proliferation, bone remodeling, leukocyte activity, and wound healing. Disturbance of mitochondrial quality control contributes to chronic inflammatory conditions, including periodontitis, cardiovascular disease, metabolic dysfunction, and tissue responses to environmental toxicants [1,2,3,4].

Hypoxia is one of the most potent physiologic regulators of mitochondrial function. Its effects are highly pattern-dependent. Brief, moderate exposures can activate adaptive signaling, including mitochondrial biogenesis, antioxidant defense, angiogenesis, and metabolic flexibility. In contrast, severe or repetitive hypoxia–reoxygenation cycles can provoke excessive production of reactive oxygen species, mitochondrial fragmentation, activation of the inflammatory pathway, and tissue injury [5,6,7].

This duality is especially relevant when comparing structured intermittent hypoxia training with the uncontrolled chronic intermittent hypoxia that accompanies obstructive sleep apnea. In athletic and rehabilitation settings, intermittent hypoxia is used deliberately to stimulate adaptation. In untreated OSA, recurrent upper-airway collapse leads to repeated desaturations, arousals, and sympathetic surges during sleep, creating a pathophysiologic pattern associated with endothelial dysfunction, insulin resistance, cardiovascular injury, periodontitis, and oral dysbiosis [8,9,10,11,12,13]. Sleep-disordered breathing symptoms are increasingly recognized as population-level markers of vascular risk [9,14], and CIH-induced insulin resistance has now been demonstrated in lean humans and lean rodents independent of obesity [15,16], implying a direct hypoxia-driven mechanism rather than mere comorbidity. Ceramide-mediated mitochondrial dysfunction is among the leading mechanistic candidates and is discussed in Section 6 below.

The aim of this review is to synthesize evidence on mitochondrial responses to cyclic altitude training and intermittent hypoxia, contrast those responses with the maladaptive CIH of OSA, and discuss the implications for the oral–airway axis. The review further integrates work on smoke-related ceramide signaling and mitochondrial injury to highlight how inhaled exposures, sleep-disordered breathing, and periodontal inflammation may converge mechanistically [17].

## 2. Methods

### Literature Search Strategy

This article was prepared as a narrative review. Literature was identified through searches of PubMed, Google Scholar, and publisher websites from database inception through May 2026 using combinations of the terms “intermittent hypoxia”, “intermittent hypoxia training”, “cyclic altitude training”, “mitochondrial biogenesis”, “PGC-1alpha”, “hypoxia-inducible factor”, “obstructive sleep apnea”, “chronic intermittent hypoxia”, “periodontitis”, “oral microbiome”, “ceramide”, “mitochondrial respiration”, and “nitric oxide”. Reference lists of identified reviews and meta-analyses were searched to capture additional relevant reports. Titles and abstracts were screened by the lead author; full texts of potentially relevant articles were retrieved and assessed against the eligibility criteria below.

Studies were included if they addressed one or more of the following: (1) intermittent hypoxia or OSA-related CIH with mitochondrial endpoints; (2) the relationship between OSA and periodontitis or oral microbiome changes; or (3) airway or toxicant biology with explicit mitochondrial, ceramide, oxidative-stress, or vascular outcomes. Studies were excluded if they were conference abstracts without accompanying peer-reviewed data, addressed hypoxia exclusively in non-mammalian systems without a translational context, or reported no primary outcome relevant to any of the three domains above. Key studies were selected to illustrate mechanistic concepts across each domain; priority was given to systematic reviews, meta-analyses, and randomized controlled trials where available and to original mechanistic studies when higher-level syntheses were unavailable. Because this is a narrative review, no formal risk-of-bias scoring was performed.

## 3. Mitochondrial Biology and Hypoxic Signaling

### 3.1. Mitochondrial Biogenesis and Quality Control

Mitochondrial homeostasis depends on coordinated biogenesis, fusion–fission dynamics, mitophagy, and proteostatic repair. PGC-1alpha serves as a master transcriptional coactivator that links environmental signals such as exercise, hypoxia, and nutrient availability to the transcriptional program required for mitochondrial biogenesis. Downstream targets include nuclear respiratory factors and mitochondrial transcription factor A, which together support mitochondrial DNA replication and respiratory protein expression [1,2,3].

Mitochondrial dynamics are regulated principally by mitofusins, optic atrophy 1, and dynamin-related protein 1. Fusion permits content exchange and network integrity, whereas fission isolates damaged mitochondrial segments for mitophagy. When these processes fail, dysfunctional mitochondria accumulate, reactive oxygen species increase, and inflammatory signaling is amplified. This framework is relevant to periodontal tissues, where oxidative injury, disordered mitophagy, and broader mitochondrial quality-control failure likely contribute to tissue destruction and impaired healing [1,2,3,18].

### 3.2. Hypoxia-Inducible Factors and Reactive Oxygen Species

Hypoxia-inducible factors orchestrate the transcriptional response to falling oxygen tension. Stabilization of HIF-alpha subunits under hypoxia shifts metabolism toward glycolysis, promotes angiogenic signaling, and alters mitochondrial oxygen consumption and substrate use. Interactions between HIF signaling and mitochondria are bidirectional: mitochondria both generate signals that influence HIF stabilization and respond to HIF-driven remodeling. Evidence for these pathways in periodontal tissues has grown considerably, and they appear central to the host–environment interface that shapes inflammatory phenotypes [5,6,19].

Reactive oxygen species are central to the distinction between adaptation and injury. Excess ROS damages lipids, proteins, and nucleic acids; however, transient, moderate ROS generation can act as a second messenger, promoting antioxidant defense and mitochondrial biogenesis. Intermittent hypoxia protocols aim to exploit this hormetic window. In inflamed periodontal tissues, ROS derives from both host immune cells and mitochondria, meaning that the local tissue response to hypoxia is shaped by both microbial challenge and mitochondrial resilience [6,19,20].

## 4. Cyclic Altitude Training and Intermittent Hypoxia Training

### 4.1. Definitions and Common Protocols

Cyclic altitude training includes several related paradigms: intermittent hypoxic exposure at rest, intermittent hypoxic training during exercise, live high–train low, live low–train high, and repeated-sprint training in hypoxia. Most normobaric protocols use inspired oxygen fractions corresponding to altitudes of 2000–4000 m, with repeated cycles of hypoxia and normoxia over several weeks [21,22].

Although protocols differ substantially across studies, the concept of the hypoxic dose is central. Adaptation depends on several interacting variables: the inspired oxygen fraction (FiO2), which in most normobaric IHT protocols falls between 10% and 16% (roughly equivalent to 2500–5000 m altitude); the length of each hypoxic bout, typically 5–7 min; the number of cycles per session, usually 4–6; the recovery interval at normoxia between bouts (3–5 min); the weekly session frequency (3–5 per week); and total program duration, which in the published literature ranges from 2 to 6 weeks. These parameters collectively determine whether a given protocol is likely to stay within a hormetic range or tip toward maladaptation [21,22].

### 4.2. Mitochondrial Adaptations to Controlled Intermittent Hypoxia

Experimental data show that appropriately dosed intermittent hypoxia can increase mitochondrial content, improve mitochondrial ultrastructure, and enhance oxidative enzyme activity (see Table 1). In rats, normobaric hypoxia combined with high-intensity intermittent training increased citrate synthase activity and upregulated proteins involved in oxidative phosphorylation, mitochondrial biogenesis, and mitochondrial fusion relative to comparable normoxic training [23].

Human evidence remains less direct, but systematic and umbrella reviews support the view that carefully designed intermittent hypoxia protocols can improve aerobic and anaerobic performance, as well as some measures of muscular strength, in both trained and untrained populations. These effects are heterogeneous and appear to depend strongly on individualization and protocol quality [21,22]. The inter-individual variability in response to IHT is substantial and likely reflects differences in several baseline physiological factors. Participants who are most likely to remain within the safe hormetic window share a cluster of favorable characteristics: a preserved hypoxic ventilatory response (HVR), which allows the respiratory system to appropriately increase ventilation when SpO2 falls; adequate baseline mitochondrial reserve, as reflected by higher pre-intervention citrate synthase activity or VO2max; low resting oxidative stress burden; and the absence of cardiovascular or pulmonary comorbidities that limit oxygen delivery or increase arrhythmic risk. Conversely, blunted HVR (more common in older adults, chronic opioid users, and some patients with obesity hypoventilation), pre-existing endothelial dysfunction, elevated baseline ceramide or inflammatory cytokine levels, and poor cardiorespiratory fitness may predispose an individual to tipping beyond the hormetic threshold into excessive ROS generation, mitochondrial fragmentation, and maladaptive inflammatory signaling. These considerations reinforce the importance of pre-participation physiological screening before IHT is prescribed outside of athletic populations [6,7,21,22].

### 4.3. Hormesis and Safety

The therapeutic promise of IHT lies in hormesis: low-to-moderate intermittent stress can trigger adaptations that make tissues more resilient to future stress. That promise also defines its main limitation. The same hypoxic load may be adaptive in a healthy, monitored participant and hazardous in an individual with significant cardiovascular, pulmonary, or arrhythmic disease. For clinicians, this means that controlled intermittent hypoxia cannot be equated with the uncontrolled CIH of OSA and should not be considered benign simply because both involve transient desaturation [6,7,21].

## 5. Obstructive Sleep Apnea, Chronic Intermittent Hypoxia, and Periodontitis

### 5.1. Pathophysiology of CIH in OSA

OSA is characterized by repetitive upper-airway collapse during sleep, leading to recurring cycles of oxygen desaturation and reoxygenation. These cycles provoke bursts of oxidative stress, activate inflammatory transcriptional pathways, increase sympathetic tone, impair endothelial function, and contribute to cardiometabolic risk [8,9,10].

Unlike therapeutic intermittent hypoxia, OSA-related CIH is not titrated, occurs during a vulnerable physiologic state, and commonly coexists with obesity, cardiometabolic disease, and smoking exposure. These contextual differences likely explain why the same broad phenomenon of hypoxia–reoxygenation can produce benefit in one setting and harm in another [8,9,10].

### 5.2. Clinical Links Between OSA and Periodontitis

Recent reviews aimed at clinicians and formal evidence syntheses have concluded that OSA and periodontal disease share inflammatory pathways, oxidative stress biology, and cardiometabolic risk factors. Available clinical literature supports an association between OSA and worse periodontal status, although causality and the magnitude of effect remain incompletely defined [11,12,24,25].

A practical interpretation is that OSA may both reflect and reinforce oral–systemic inflammatory burden. Conversely, chronic periodontal inflammation may amplify systemic inflammatory signaling in patients already exposed to CIH. These observations support closer collaboration between dental clinicians, sleep physicians, and primary care teams [10,11,12,24,25].

### 5.3. Oral Microbiome Changes in OSA and Periodontitis

The oral microbiome is shaped by oxygen tension, salivary flow, pH, host immune activity, and local mechanical factors. Oxygen availability is a particularly strong driver: within established periodontal pockets, oxygen tension can fall below 1%, creating a strongly anaerobic microenvironment that enriches for obligate anaerobes—*Porphyromonas gingivalis*, *Treponema denticola*, *Tannerella forsythia* (the classic periodontal red complex), and *Fusobacterium nucleatum*—while suppressing the commensal aerotolerant streptococci and Veillonella species that characterize healthy sulci. Li et al. found that OSA alone, periodontitis alone, and combined OSA plus periodontitis each produce distinct salivary microbiome profiles, with progressive shifts in alpha and beta diversity across these groups [13]. The specific taxa driving those shifts were not identical across conditions, which suggests the diseases act through at least partially independent mechanisms rather than simply summing their effects (see Table 2).

The oxygen-tension mechanism deserves some attention here. CIH in OSA produces fluctuating systemic hypoxia throughout the night—typically dozens of desaturation events, sometimes with SpO2 nadirs below 80% in severe cases—and this systemic perturbation may compound the already low-oxygen microenvironment of diseased periodontal pockets. Whether OSA-related hypoxia directly deepens anaerobic selection in the subgingival niche beyond what periodontitis alone would produce remains unanswered in the existing literature. Single-organism studies show that *P. gingivalis* thrives under strict anaerobiosis and that its gingipain virulence factors are maximally expressed under low-oxygen conditions. A reasonable working hypothesis is that OSA adds a systemic hypoxic gradient that tips marginally dysbiotic communities toward more anaerobic, tissue-destructive configurations—but this remains to be demonstrated with WGS-level microbiome data in sleep apnea cohorts. Prospective cohorts linking polysomnographic OSA severity metrics (apnea–hypopnea index, oxygen desaturation index, time spent below SpO2 90%) with longitudinal shotgun metagenomic sequencing of subgingival plaque offer the most direct path to disentangling the contribution of systemic nocturnal hypoxia from the confounding effect of concurrent periodontitis progression. WGS-resolution data would enable tracking of strain-level shifts and accessory genome changes that amplicon-based surveys cannot resolve, and could reveal whether hypoxia-responsive virulence genes in keystone pathogens are upregulated specifically in patients with a high nocturnal desaturation burden, independent of pocket depth. Studies using paired baseline and 12-month follow-up sampling, with OSA treatment (CPAP or mandibular advancement) as an intervention arm, would be especially informative: if CPAP-mediated restoration of nocturnal normoxia attenuates dysbiotic drift detectable at the WGS level, that would provide strong evidence that OSA-specific hypoxia independently shapes the subgingival ecosystem beyond what periodontitis alone explains.

### 5.4. Mitochondrial and Vascular Mechanisms Relevant to Periodontal Tissues

Periodontal tissues are highly vascularized and metabolically active. CIH-related endothelial dysfunction may reduce microvascular efficiency, while excessive mitochondrial and leukocyte-derived ROS can promote collagen breakdown, osteoclastogenesis, and delayed healing. Hypoxia signaling within periodontal pockets may initially support adaptive angiogenesis, but under chronic inflammatory conditions, the balance can shift toward maladaptive tissue remodeling. Disturbed mitophagy and broader mitochondrial quality-control failure further amplify inflammatory injury in periodontitis, pointing to mitochondria as a likely mechanistic link between OSA-related hypoxia and periodontal breakdown [9,10,18,19,20,24,25]. Ceramide accumulation in periodontal tissues—driven by both local oxidative stress and systemic CIH or inhaled toxicant exposure—provides a specific lipid-signaling explanation for the mitochondrial fragmentation, NLRP3 inflammasome activation, and impaired healing observed in periodontitis [4,10,26].

## 6. Ceramide–Mitochondrial Crosstalk: A Sphingolipid Bridge Between Hypoxia, Inhaled Toxicants, and Insulin Resistance

### 6.1. Ceramide Accumulation as a Hypoxia-Responsive Lipid Signal

Sphingolipid metabolism responds directly to cellular oxygen tension. The de novo ceramide biosynthesis pathway terminates with dihydroceramide desaturase (DEGS1), an oxygen-dependent reaction that introduces the canonical 4,5-trans double bond in the ceramide backbone. Under hypoxia, DEGS1 activity is suppressed, shifting the sphingolipid pool toward bioactive dihydroceramides and altering downstream signaling [27,28]. In parallel, both hypoxia–reoxygenation and inhaled toxicant exposure can activate sphingomyelinases and serine palmitoyltransferase, the rate-limiting enzyme in de novo ceramide synthesis [29]. The net consequence in CIH and chronic hypoxia models is increased ceramide and dihydroceramide content in the liver, vascular endothelium, and adipose tissue [10,16,30], paralleling the smoke-induced accumulation of ceramide in murine cardiomyocytes previously documented [4]. That CIH and inhaled toxicants converge on the same lipid-signaling pathway helps explain the airway-systemic injury patterns reviewed above.

### 6.2. Mitochondrial Fission and Respiratory Dysfunction as the Downstream Consequence

Ceramides are not bystanders in mitochondrial physiology; they are active drivers of structural and functional remodeling. In skeletal muscle, ceramide accumulation triggers DRP1-mediated mitochondrial fission, fragmenting the network and impairing oxidative respiration; pharmacologic inhibition of fission with Mdivi-1 rescues this phenotype, identifying mitochondrial dynamics as the proximate mechanism rather than a downstream correlate [26]. The same lipid-driven fission program operates in cardiomyocytes, where myriocin-mediated ceramide blockade preserves both mitochondrial respiration and insulin sensitivity [31]. Mechanistically, ceramides interact with the outer mitochondrial membrane, disrupt fusion machinery, inhibit electron transport chain intermediates, and amplify reactive oxygen species generation [29]. This is consistent with several findings already cited in this review: the smoke-induced impairment of cardiomyocyte mitochondrial respiration [4], the smoke-induced systemic metabolic disruption mediated by ceramides in vivo [17], and the CIH-induced TXNIP/NLRP3 endothelial injury [10] all converge on ceramide-driven mitochondrial fragmentation as a shared proximate lesion.

### 6.3. From Mitochondrial Dysfunction to Insulin Resistance

Ceramide accumulation impairs insulin signaling through at least two well-characterized mechanisms: protein phosphatase 2A (PP2A)-mediated dephosphorylation of Akt and PKC-zeta-mediated sequestration of Akt away from the plasma membrane, both of which block downstream GLUT4 translocation and glucose uptake [29,32]. These effects are not confined to obesity or chronic overnutrition. Recent human and rodent CIH studies show that intermittent hypoxia alone—in lean, non-obese subjects—reproducibly induces systemic insulin resistance, elevated free fatty acid flux, hepatic mitochondrial dysfunction, and defects in adipose tissue insulin signaling [15,16,33]. The temporal co-occurrence of elevated ceramides, mitochondrial fission, and impaired insulin signaling in CIH models is consistent with the mechanistic sequence proposed here, whereby CIH elevates ceramide levels, ceramides promote DRP1-mediated fission and ROS generation, and the resulting mitochondrial dysfunction may impair insulin signaling and oral–systemic vascular function, but direct causal evidence in humans remains limited. For the dental and airway-focused clinician, these data suggest that insulin resistance in OSA may, at least in part, reflect a hypoxic-lipid signaling contribution rather than obesity alone, though prospective intervention studies are needed to test this hypothesis in clinical populations.

## 7. Airway Biology, Environmental Exposures, and Mitochondrial Dysfunction

### 7.1. Ceramides, Smoke Exposure, and Mitochondrial Respiration

Research by Reynolds and colleagues directly bears on the field of oral–airway management. In mouse models, cigarette smoke exposure increased ceramide accumulation and disrupted metabolism, while related work demonstrated cardiomyocyte ceramide accumulation and impaired mitochondrial respiration. These studies show that inhaled toxicants can drive mitochondrial dysfunction through lipid signaling pathways, not merely through nonspecific oxidative stress [4,17].

For dentistry and airway-focused practice, this is important because smoke and other inhaled toxicant exposures are unlikely to affect only the lungs. Oral epithelial surfaces, salivary tissues, and periodontal structures share vulnerability to oxidative and mitochondrial injury, suggesting that environmental exposure history should be integrated into oral–systemic risk assessment [1,4,17].

### 7.2. Translational Implications for the Oral–Airway Axis

Airway obstruction, CIH, and inhaled toxicants likely act on overlapping pathways—mitochondrial dysfunction, altered redox balance, epithelial injury, ceramide signaling, and vascular dysregulation. That overlap goes some way toward explaining why patients with OSA, smoking exposure, and periodontal inflammation tend to present with a compounded oral–systemic risk profile rather than three independent problems. A mitochondrial framing can help clinicians see what those conditions share [8,9,10,17].

## 8. Can Intermittent Hypoxia Training Become an Adjunct in OSA and Periodontal Care?

### 8.1. Conceptual Framework

The most logical translational model is not to use intermittent hypoxia as an alternative to OSA treatment but to consider whether carefully monitored IHT could serve as an adjunct only after effective control of pathologic nocturnal CIH. In that context, the goal would be to activate adaptive mitochondrial signaling without recreating the harmful hypoxic pattern seen in untreated sleep apnea [7,8,24,25].

### 8.2. Potential Benefits and Current Limitations

Post-treatment IHT could, in principle, improve vascular function and exercise tolerance in patients who have achieved durable OSA control. Whether those effects translate into meaningful periodontal outcomes—reduced tissue inflammation, improved wound healing after periodontal therapy, and a lower systemic inflammatory burden—remains unknown. These are reasonable questions to ask, but they remain questions [21,22,23,34].

The safety question deserves more than a passing caveat. The typical OSA patient who might be considered for adjunct IHT is not a healthy athlete. This population commonly carries hypertension, type 2 diabetes, dyslipidemia, and, in a substantial fraction of cases, established coronary artery disease or a history of atrial fibrillation. Deliberately inducing hypoxia in these patients—even at the moderate FiO2 levels used in therapeutic protocols—is not a low-risk proposition. Arrhythmia provocation, demand ischemia, and exacerbation of pulmonary hypertension are real concerns that have not been systematically evaluated in IHT trials, because most of those trials excluded exactly this kind of patient. The existing IHT literature primarily draws on trained athletes, healthy sedentary adults, or highly screened patient populations; these groups are not representative of a typical sleep clinic referral. Until prospective safety data are available in OSA patients with common comorbidities, IHT in this setting should be considered experimental and reserved for patients who have been formally screened for arrhythmia risk, uncontrolled hypertension, and impaired cardiorespiratory reserve [7,8,21].

### 8.3. Limitations

Several limitations of this narrative review warrant explicit acknowledgment. First, because this is a narrative rather than a systematic review, no formal risk-of-bias assessment was conducted; studies were selected to illustrate mechanistic concepts, and publication bias toward positive findings cannot be excluded. Second, direct human evidence for mitochondrial endpoints following IHT is limited; most mechanistic data come from rodent models, and extrapolation to clinical populations must be made with caution. Third, IHT protocols differ substantially across the reviewed studies in terms of FiO2 (10–16%), bout duration (5–7 min), number of cycles per session (4–6), recovery intervals (3–5 min), weekly session frequency (3–5 sessions), and total program duration (2–6 weeks); this heterogeneity limits cross-study comparisons and makes it difficult to define an optimal hypoxic dose for mitochondrial benefit. Fourth, the mechanistic pathway from CIH to ceramide accumulation to insulin resistance, while biologically plausible and supported by converging experimental evidence, has not been confirmed in prospective human trials with prespecified ceramide endpoints; causal language in this review reflects mechanistic hypotheses rather than established clinical facts. Fifth, the association between OSA and periodontal disease, though supported by systematic reviews, remains incompletely characterized with respect to the direction of effect, the magnitude of attributable risk, and confounding by shared risk factors.

### 8.4. Research Priorities

Future trials should focus on patients with effectively treated OSA and well-characterized periodontal status. Priority outcomes include clinical periodontal measures, endothelial function, salivary and serum inflammatory biomarkers, oxidative-stress markers, and patient-reported quality-of-life measures. Mechanistic studies using peripheral blood mononuclear cells, gingival fibroblasts, or buccal cells would be especially valuable for determining whether systemic hypoxic conditioning produces measurable mitochondrial effects in the oral cavity [13,24,25,34].

## 9. Clinical Implications for Dental and Airway-Centric Practice

### 9.1. Screening and Co-Management

Dental teams are well-positioned to identify patients who may benefit from a sleep evaluation. Periodontal inflammation, mouth breathing, craniofacial risk patterns, obesity, cardiometabolic disease, and smoking exposure should prompt consideration of OSA screening and interdisciplinary referral. Conversely, medical sleep clinicians should recognize that the oral cavity may provide visible evidence of systemic inflammatory burden [8,24,25].

### 9.2. Nitric Oxide and Clinical Implications

Nitric oxide (NO) is produced in exceptional quantities by the paranasal sinuses and nasal mucosa, where constitutive nitric oxide synthase activity generates luminal NO concentrations that are far higher than those measured in exhaled lower-airway air. During nasal breathing, this NO reservoir is entrained into the inhaled airstream and conveyed to the bronchial tree and pulmonary vasculature, where it acts as a bronchodilator, vasodilator, and antimicrobial effector [35].

At the mitochondrial level, NO exerts concentration-dependent and bidirectional effects on the respiratory chain. At physiologic nanomolar concentrations, NO reversibly inhibits cytochrome c oxidase in competition with molecular oxygen, while endothelial NO signaling can also drive mitochondrial biogenesis through cGMP-dependent activation of PGC-1alpha [36,37].

Research on human dietary nitrate provides functional corroboration of these mechanistic findings. Oral supplementation with inorganic nitrate can lower the oxygen cost of submaximal exercise and improve mitochondrial coupling efficiency, consistent with NO-mediated optimization of respiratory-chain function. In the context of the airway axis reviewed here, this raises the question of whether chronic mouth breathing and nasal obstruction remove a meaningful source of NO delivery, thereby reducing the pulmonary, vascular, and mitochondrial signaling that nasal breathing provides [35,36,37,38,39].

### 9.3. Mitochondria-Aware Preventive Strategies

Several practical interventions are already consistent with a mitochondrial approach to oral–systemic health: regular physical activity, smoking cessation, reduction in inhaled toxicant exposure, dietary patterns that support glycemic control and antioxidant capacity, and effective treatment of sleep-disordered breathing. Even if intermittent hypoxia remains experimental in this population, mitochondrial-supportive lifestyle measures are immediately actionable [1,2,8,17,21,24,25].

## 10. Conclusions

Hypoxia is not inherently beneficial or harmful; its biologic impact depends on dose, timing, pattern, and clinical context. Controlled intermittent hypoxia can promote mitochondrial adaptation and performance-related benefits, whereas OSA-related chronic intermittent hypoxia drives oxidative stress, inflammation, vascular dysfunction, and oral–systemic injury [6,7,8,9,10,21,22,23,24,25].

For clinicians working at the intersection of dentistry, airway health, and systemic medicine, mitochondria offer a coherent conceptual framework linking periodontal disease, sleep-disordered breathing, environmental exposures, and cardiometabolic risk. The next step is translational: well-designed trials are needed to determine whether adaptive hypoxic conditioning has a role as a carefully controlled adjunct after effective management of pathologic hypoxia in OSA [1,2,13,17,24,25,34]. Across these contexts, ceramide-mediated mitochondrial dysfunction represents a mechanistically plausible unifying hypothesis connecting CIH, inhaled toxicant exposure, periodontal inflammation, and systemic insulin resistance; if confirmed in prospective human studies, it would identify the sphingolipid pathway as a tractable target for future translational and pharmacologic research.

## Figures and Tables

**Table 1:** Representative IHT protocols: populations, hypoxic dose parameters, and mitochondrial or performance outcomes.

Study; Population; Hypoxic Dose	Key Mitochondrial/Performance Outcome
Dobashi et al. 2024 [23]; rats (gastrocnemius); normobaric; ~13%; HIIT-matched bouts; 5/wk; 4 wk	↑ citrate synthase; ↑ PGC-1α, OPA1 vs. normoxic HIIT; improved mitochondrial biogenesis and dynamics
Boulares et al. 2025 [21]; umbrella review; trained and untrained humans; mixed normobaric/hypobaric; FiO2 10–16%; 5–7 min bouts; 4–6 cycles; 3–5/wk; 2–6 wk	↑ VO2max; improved aerobic and anaerobic performance; high inter-protocol heterogeneity; no direct biopsy mitochondrial data
Huang et al. 2023 [22]; meta-analysis; exercisers (mixed fitness); normobaric; FiO2 12–15%; variable bouts/cycles; 3–8 wk	↑ VO2max; improved aerobic capacity; substantial heterogeneity; no biopsy mitochondrial data
Briaçon-Marjollet et al. 2025 [15]; crossover RCT; healthy lean humans; simulated OSA-pattern nocturnal CIH; FiO2~13%; ~30 events/h; 2 wk	Induced insulin resistance; ↑ free fatty acid flux; lipid dysregulation independent of obesity; maladaptive CIH phenotype

Abbreviations: CIH, chronic intermittent hypoxia; FFA, free fatty acid; FiO2, fraction of inspired oxygen; HIIT, high-intensity interval training; RCT, randomized controlled trial; VO2max, maximal oxygen uptake. ↑, increase.

**Table 2:** Summary of evidence linking OSA, periodontitis, oral microbiome changes, and mitochondrial or inflammatory endpoints.

Study; Type; Endpoints	Key Finding
Lembo et al. 2021 [11]; systematic review; PSG-confirmed OSA; Periodontal disease severity; oxidative stress and inflammatory markers (indirect)	OSA associated with worse periodontal status; shared inflammatory and oxidative pathways proposed; causality not established
Khodadadi et al. 2022 [12]; meta-analysis; AHI (PSG); Periodontal disease (clinical attachment loss); not assessed directly	OSA patients had significantly higher odds of periodontitis (OR~2.4); high heterogeneity; and confounding by BMI and smoking acknowledged
Li et al. 2025 [13]; cross-sectional; PSG-confirmed OSA; Salivary microbiome (16S rRNA); periodontal status; inflammatory cytokine profiles (indirect via dysbiosis)	Distinct salivary microbiome shifts in OSA-alone, periodontitis-alone, and combined groups; progressive ↓ alpha diversity; taxa driving shifts are partially independent between conditions
Yan et al. 2021 [10]; animal model (rat CIH); vascular endothelial function; mitochondrial dysfunction; TXNIP/NLRP3/IL-1β axis; ROS	CIH-induced mitochondrial dysfunction mediates endothelial injury via TXNIP/NLRP3 inflammasome; identifies mitochondria as a mechanistic bridge to vascular damage
Carra & Cistulli 2024 [24]; Incerti Parenti et al. 2025 [25]; clinical reviews; OSA; periodontal disease; oral–systemic links; IL-6, TNF-α, CRP	Both conditions amplify systemic inflammatory and cardiometabolic burden; interdisciplinary screening is recommended

Abbreviations: AHI, apnea–hypopnea index; CIH, chronic intermittent hypoxia; CRP, C-reactive protein; IL, interleukin; NLRP3, NLR family pyrin domain containing 3; OSA, obstructive sleep apnea; PSG, polysomnography; ROS, reactive oxygen species; TNF-α, tumor necrosis factor alpha; TXNIP, thioredoxin-interacting protein. ↓, decrease.

## Data Availability

No new data were created or analyzed in this study. Data sharing is not applicable to this article.
